# Lack of chronic neuroinflammation in the absence of focal hemorrhage in a rat model of low-energy blast-induced TBI

**DOI:** 10.1186/s40478-017-0483-z

**Published:** 2017-11-10

**Authors:** Miguel A. Gama Sosa, Rita De Gasperi, Georgina S. Perez Garcia, Heidi Sosa, Courtney Searcy, Danielle Vargas, Pierce L. Janssen, Gissel M. Perez, Anna E. Tschiffely, William G. Janssen, Richard M. McCarron, Patrick R. Hof, Fatemeh G. Haghighi, Stephen T. Ahlers, Gregory A. Elder

**Affiliations:** 1 General Medical Research Service, James J. Peters Department of Veterans Affairs Medical Center, 130 West Kingsbridge Road, Bronx, New York, 10468 USA; 2 Research and Development Service, James J. Peters Department of Veterans Affairs Medical Center, Bronx, New York, USA; 3 Neurology Service, James J. Peters Department of Veterans Affairs Medical Center, Bronx, New York, USA; 4 Department of Psychiatry, Icahn School of Medicine at Mount Sinai, New York, NY USA; 5 Department of Neurology, Icahn School of Medicine at Mount Sinai, New York, NY USA; 6 Fishberg Department of Neuroscience, Icahn School of Medicine at Mount Sinai, New York, NY USA; 7 Department of Geriatrics and Palliative Care, Icahn School of Medicine at Mount Sinai, New York, NY USA; 8 Friedman Brain Institute, Icahn School of Medicine at Mount Sinai, New York, NY USA; 9 Operational and Undersea Medicine Directorate, Naval Medical Research Center, Silver Spring, MD USA; 10 Department of Surgery, Uniformed Services University of the Health Sciences, Bethesda, MD USA

## Abstract

Blast-related traumatic brain injury (TBI) has been a common cause of injury in the recent conflicts in Iraq and Afghanistan. Blast waves can damage blood vessels, neurons, and glial cells within the brain. Acutely, depending on the blast energy, blast wave duration, and number of exposures, blast waves disrupt the blood-brain barrier, triggering microglial activation and neuroinflammation. Recently, there has been much interest in the role that ongoing neuroinflammation may play in the chronic effects of TBI. Here, we investigated whether chronic neuroinflammation is present in a rat model of repetitive low-energy blast exposure. Six weeks after three 74.5-kPa blast exposures, and in the absence of hemorrhage, no significant alteration in the level of microglia activation was found. At 6 weeks after blast exposure, plasma levels of fractalkine, interleukin-1β, lipopolysaccharide-inducible CXC chemokine, macrophage inflammatory protein 1α, and vascular endothelial growth factor were decreased. However, no differences in cytokine levels were detected between blast-exposed and control rats at 40 weeks. In brain, isolated changes were seen in levels of selected cytokines at 6 weeks following blast exposure, but none of these changes was found in both hemispheres or at 40 weeks after blast exposure. Notably, one animal with a focal hemorrhagic tear showed chronic microglial activation around the lesion 16 weeks post-blast exposure. These findings suggest that focal hemorrhage can trigger chronic focal neuroinflammation following blast-induced TBI, but that in the absence of hemorrhage, chronic neuroinflammation is not a general feature of low-level blast injury.

## Introduction

Military personnel exposed to blast overpressures are at risk of developing behavioral and cognitive abnormalities [11]. Acutely, several studies in animal models have shown that blast exposure induces brain inflammation and increased levels of pro-inflammatory factors. Infiltration of polymorphonuclear leukocytes and lymphocytes is observed in the brain parenchyma within 1 h post-blast exposure [53]. In rats, a single 551-kPa blast exposure induced glial fibrillary acidic protein (GFAP), S100β (an astrocytic marker), cyclooxygenase (COX)-2, interleukin (IL)-1β, and tumor necrosis factor (TNF)-α, and these changes were present by 1 h and remained detectable at 3 weeks post-injury [53]. Similarly, GFAP levels were significantly higher in all measured brain regions of rats exposed to 133.8-kPa blast overpressure [26]. In another study, a 120-kPa blast triggered release of IL-1β, TNF-α, IL-10, and erythropoietin (EPO) as early as 3 h after injury, and peak levels were reached at 24 h before they diminished by 48 h [33]. Moreover, a 129-kPa blast induced transcriptional upregulation of the proinflammatory interferon (IFN)-γ and monocyte chemoattractant protein (MCP)-1 by 4 h post-blast, and increased levels of these proteins were detected 24 h post-blast while strong Iba1-immunoreactive microglial cell activation was detected at 2 weeks post-blast [8]. Elevated levels of IFN-γ and IL-6 have also been found in the amygdala and ventral hippocampus, together with an increased number of Iba1-expressing activated microglia following blast injury [26].

A cDNA microarray analysis of the brains of mice exposed to a 142-kPa blast showed significant upregulation of several interleukin receptors in the midbrain [55]. In the frontal cortex, hippocampus, and cerebellum, the expression of IL receptors was significantly reduced, whereas the expression of TNF-α and its receptors was increased [55]. In rats subjected to multiple 138-kPa blast exposures (5×), plasma levels of vascular endothelial growth factor (VEGF) and neuron-specific enolase (NSE) were significantly elevated after 2 h compared to levels in sham controls and rats exposed to a single blast [27]. By day 22 post-injury, animals exposed to either a single blast or multiple blasts had significantly higher levels of VEGF, neuron-specific enolase (NSE), neurofilament H (NFH), and GFAP than did those in the non-injured control groups [27]. At 1 day post-blast exposure, increased GFAP immunoreactivity was observed in the hippocampus of animals exposed to a single blast, whereas no such increase was seen in animals exposed to multiple blasts [27]. However, by day 22, an apparent increase in GFAP immunoreactivity was observed in the hippocampus of the multiple blast-exposed rats. In both the single and multiple blast-exposed groups, increased apoptosis was found in the hippocampal hilus [27] .

In a temporal evaluation of cytokines in rat serum after a single 117-kPa blast exposure, a significant decrease in IL-1α expression was observed at 3 h as well as a decrease in M-CSF expression at 24 h, an increase in EPO at 48 h, and decreased levels of IL-1α, IL-1β, IL-6, IL-10, and EPO along with increased levels of VEGF and macrophage colony stimulating factor (M-CSF) at 72 h post-blast [44]. Blast injury (142 kPa) in stressed rats resulted in elevated levels of serum corticosterone, NFH, NSE, GFAP, and VEGF compared to levels in non-blast-exposed controls 2 months post-injury. The hippocampus and prefrontal cortex (PFC) also contained increased numbers of apoptotic cells (TUNEL-positive) and elevated levels of GFAP, S100β, Iba1, VEGF, IL-6, IFN-γ, and phosphorylated tau [32]. In humans, 35- to 81-kPa blast exposures result in increased levels of IL-6 and TNF-α in the serum that were maintained for 24 h [18].

These data altogether suggest that acute blast exposure induces brain inflammation and increased levels of pro-inflammatory factors. Acute inflammation has long been considered a transient phenomenon in many forms of TBI. However, there is accumulating evidence that the inflammatory response after TBI may persist [14]. Indeed, studies in animals document persistent inflammation in brain after TBI [14]. Postmortem human studies find inflammatory changes that can persist for years after TBI [24], and a recent study using positron emission tomography to image a ligand that targets microglia found increased microglial activation up to 17 years after injury [43] .

We previously reported that rats exposed to low-level blast overpressures (74.5 kPa) develop a post-traumatic stress disorder (PTSD)-like phenotype associated with chronic brain vascular degeneration and rarely hemorrhagic brain cortical tears that follow the lines of penetrating vessels [17, 49]. In the present study, we investigated whether three 74.5-kPa blast exposures can induce chronic microglial activation and increase the levels of pro-inflammatory factors in brain. Our results show that under this blast protocol and in the absence of vascular disruption, low-level blast exposures do not alter the microglial cell density nor microglial activation in the hippocampus and prelimbic cortex and do not significantly alter the levels of cytokines involved in neuroinflammation (over 6–40 weeks post-blast). However, in the presence of focal hemorrhage, neuroinflammation with increased microglial activation and astrocytosis was observed 16 weeks post-blast exposure. These results show that neuroinflammation is induced by leakage of blood elements from a fragile vasculature into the brain parenchyma. However, in the absence of hemorrhage, chronic neuroinflammation is not a general feature of low-level blast injury.

## Materials and methods

### Animals and blast exposure

All studies were approved by the Institutional Animal Care and Use Committees of the James J. Peters VA Medical Center, Bronx, NY and the Walter Reed Army Institute of Research/Naval Medical Research Center, Silver Spring, MD. Long Evans rats were exposed to three 74.5-kPa (10.8 psi) blasts of compressed air in a shock tube under isoflurane anesthesia at 10 weeks of age as previously described [1]. Rats were randomly assigned to sham or blast conditions with the head facing the blast exposure without any body shielding resulting in a full body exposure to the blast wave. One exposure per day was administered for three consecutive days. Control animals were anesthesized and placed inside the shock tube but not exposed to blast overpressures. For stereology, animals (*n* = 5/group) were euthanized by cardiac perfusion 6 weeks post-blast exposure with cold 4% paraformaldehyde in phosphate-buffered saline (PBS). For cytokine analyses, blast-exposed and control animals were euthanized with CO_2_ at 6 and 40 weeks post-blast exposure.

### Quantitative morphometric analyses of microglial phenotypes in the hippocampus and prelimbic cortex

Vibratome-cut coronal sections (50 μm-thick) from 4% paraformaldehyde-perfused brains of blast-exposed and control animals were sampled every 500 μm throughout the brain. Sections were rinsed with PBS, blocked with 50 mM Tris–HCl, pH 7.6, 0.15 M NaCl, 0.1% Triton X-100, and 5% goat serum (TBS-TGS) for 1 h, and incubated overnight with rabbit anti-Iba1 antibodies (ionized calcium-binding adapter molecule 1 [41], 1:300, Wako, Japan) in TBS-TGS at room temperature. After 6 washes with PBS over a period of 1 h, sections were incubated with goat anti-rabbit horseradish peroxidase-conjugated secondary antibodies (1:300, Pierce, Waltham, MA) for 2 h in TBS-TGS and rinsed with PBS. Microglial immunostaining was visualized by incubation with 0.05% 3,3′-diaminobenzidine (DAB), 0.015% H_2_O_2_ in 0.15 M NaCl, 50 mM imidazole, pH 7.0 at room temperature. Stereology of prelimbic (interaural, 12.00 mm) and hippocampal microglia (interaural, 4.44–5.76 mm) was performed using the MBF StereoInvestigator software with the Optical Fractionator probe (Williston, VT) [38]. Stereologic analyses were done with the investigator blinded to the experimental condition. Areas of interest were traced using an Olympus PlanFI 4×/0.13 objective lens on an Olympus BX51 microscope, and a PlanApo 60×/1.40 oil-immersion objective lens was used for cell counting. Sampling grids of 500 × 400 μm were randomly placed over the hippocampal and prelimbic cortical regions, and contained an optical disector of 80 × 80 μm, within which cell numbers were counted. A 1-μm guard zone was set at the top and bottom of each section. Microglial profiles contained either within the frame or touching the permitted green lines were counted, whereas those that touched the forbidden red margins were excluded. The morphology, according to the microglial phenotype, of each counted cell was also noted (types 1–4: ramified, primed, reactive, and ameboid) [30, 31, 46, 48, 50, 54]. Analysis of microglia activation in the hippocampus was further performed by immunohistochemical determination of MHC class II antigen (MHCII) expression in Iba1-positive cells. Sections (interaural, 5.64 mm) from the 5 blast-exposed and 5 control animals described above were immunostained with rabbit anti-Iba1 and mouse anti-rat MHC class II antibodies (1:200, Novus Biologicals, Littleton CO, USA) as described above. Immunostaining was detected with species-specific AlexaFluor 488- and 568-conjugated secondary antibodies (1:300; Molecular Probes, Eugene OR, USA). Nuclei were counterstained with 1 μg/ml 4′,6-diamidino-2-phenylindole (DAPI). The relative fraction of MHCII^+^/Iba1^+^ cells was determined in both hemispheres.

### Immunoassay for selected rat cytokines

Levels of selected cytokines in plasma and regional brain extracts taken from control and blast-exposed animals at 6 and 40 weeks post-blast exposure were measured (*n* = 5/group). Brains were regionally dissected and extracts were prepared from the left and right posterior cortex (association, auditory, visual, and entorhinal cortices), anterior cortex (prefrontal, motor, somatosensory, and insular cortices), hippocampus, and amygdala. The tissues were homogenized in a solution of 0.1 M Tris HCl, pH 7.6, 0.15 M NaCl, 5 mM EDTA, 0.1% sodium dodecyl sulfate (SDS), and 1% Triton X-100 supplemented with a protease and phosphatase inhibitor cocktail (Abcam, Cambridge, MA). The homogenates were centrifuged at 14,000×g for 20 min at 4 °C, and the supernatants collected for analysis. The total protein concentration was determined with the BCA reagent (Pierce, Waltham, MA), and the protein concentration of the brain samples was adjusted to 1 μg/μl. The levels of the selected cytokines/chemokines were determined using a multiplexed bead-based immunoassay [23], which has been extensively used for the study of cytokine dysregulation in rat models of blast-induced TBI [33, 44, 58] and allows for simultaneous detection of cytokines/chemokines involved in inflammation. The Milliplex MAP Rat Cytokine/Chemokine Magnetic Bead Panel-Premixed 2 (EMD Millipore, Billerica, MA) was used in our study to analyze 27 targets namely IFN-γ, interleukins IL-1α, IL-1β, IL-2, IL-4, IL-5, IL-6, IL-7, IL-10, IL-12p70, IL-13, IL-17A and IL-18, IFN γ-induced protein 10 (IP-10), leptin, lipopolysaccharide-inducible CXC chemokine (LIX), M-CSF, MCP-1, macrophage inflammatory protein (MIP)-1α and MIP-2, RANTES (Regulated on Activation, Normal T-Cell Expressed and Secreted), TNF-α, EPO, VEGF, epidermal growth factor (EGF), C-C motif chemokine 11 (eotaxin/CCL11), chemokine C-X-C motif ligand 1 (CXCL1/GRO/KC), and fractalkine.

### Statistical analyses

Between group comparisons were made using unpaired Student’s *t* tests and are reported without correction for multiple comparisons and after a Bonferroni correction for multiple comparisons. Statistical tests were performed using the program GraphPad Prism 7.0 (GraphPad Software, San Diego, CA, USA) or SPSS 24.0 (SPSS, Chicago, IL, USA).

## Results

### Similar microglial activation in control and blast-exposed animals

**Fig. 1:**
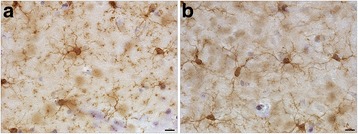
Three low-level 74.5-kPa blast exposures do not result in microglial activation. Hippocampal microglia in Vibratome-cut sections visualized by Iba1-peroxidase immunohistochemistry as described. Control (**a**); blast (**b**). Scale bar, 10 μm

Brains of 16 week-old rats were analyzed 6 weeks post-blast exposures, a time by which chronic inflammation should be well established. No evidence of the presence of hemorrhages was observed on freshly-cut sections nor on hematoxylin-eosin (HE)-stained sections. General microscopic observations of Iba1-immunoreactive cells throughout the brain did not reveal major differences in the microglial cell density or phenotype morphologies between sequential brain sections from control and blast-exposed animals and did not identify focal regions of microgliosis (Fig. 1).

**Fig. 2:**
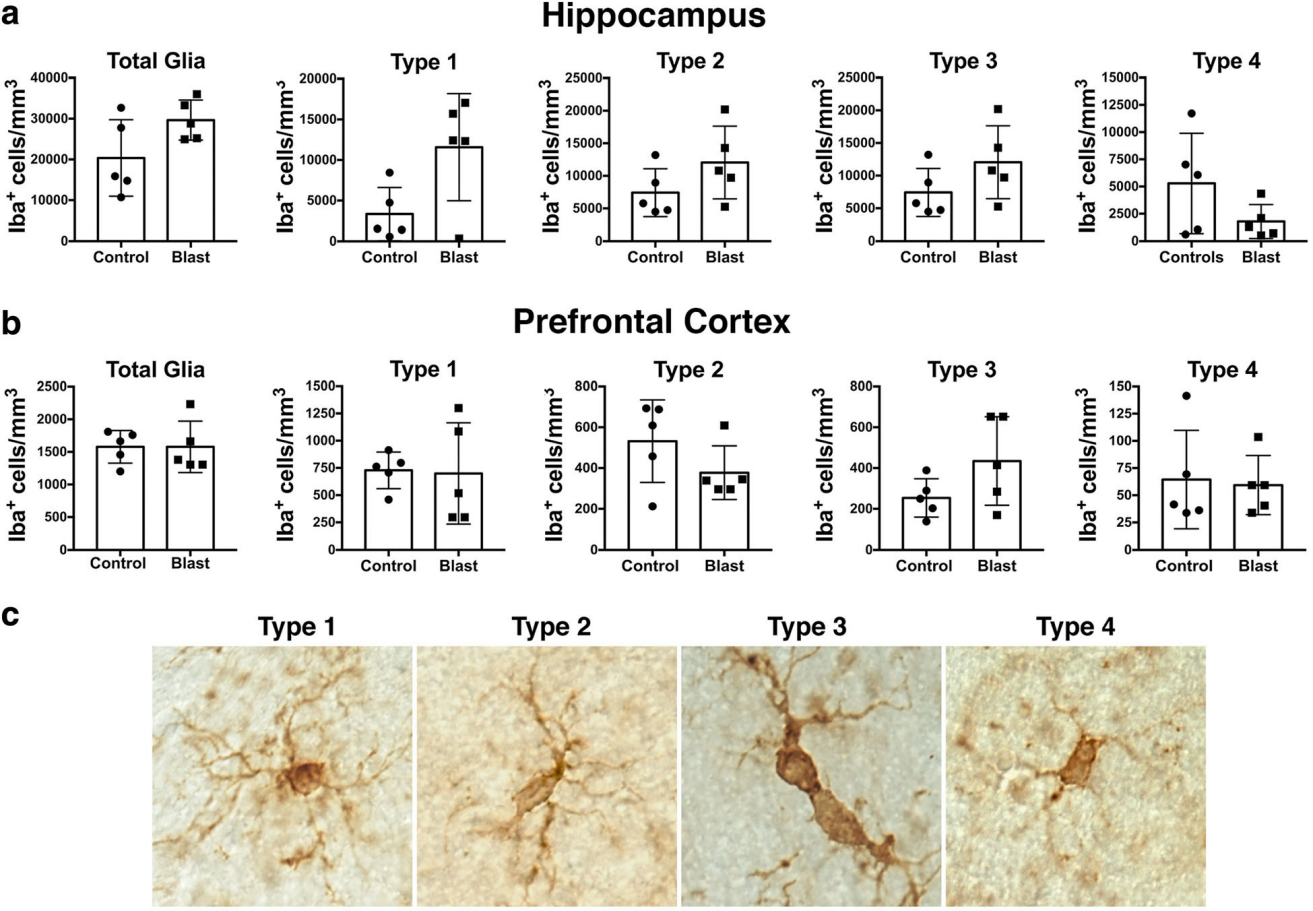
Similar local densities of microglia and microglial subtypes in the hippocampus and prefrontal cortex 6 weeks following blast exposure. Estimated densities of total microglia and microglial subtypes are shown for the hippocampus (**a**) and prefrontal cortex (**b**). Panel (**c**) shows examples of microglial subtypes. Error bars indicate the standard error of the mean (SEM). There was no statistically significant difference between blast and control

A quantitative stereologic analysis of Iba1-immunolabeled microglia was performed to evaluate the relative distribution and abundance of microglial phenotypes in the prefrontal cortex and hippocampus of blast-exposed and control animals (Fig. 2). Distinct morphological phenotypes were observed in the selected areas corresponding to the previously described microglial phenotypes associated with different states of activation including ramified, primed, reactive, and ameboid microglia (types 1–4, respectively; Fig. 2) [30, 31, 46, 48, 50, 54]. No statistically significant differences were observed in the total microglial populations in the analyzed brain regions of control and blast-exposed animals. Similarly, no significant differences were observed in the relative numbers of microglial subtypes, with the most abundant being the ramified (type 1) and primed (type 2) microglia (Figs. 1 and 2).

**Fig. 3:**
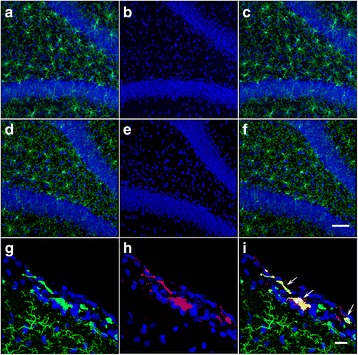
Lack of activated proinflammatory Iba1^+^ MHCII^+^ microglia in the hippocampus of blast-exposed animals (6 weeks post-blast exposure). Iba1, green; MHCII, red; DAPI, blue. Similar negligible presence of MHCII^+^ microglia (<1% of Iba1^+^ cells) was observed in the hippocampus of blast exposed (**a**-**c**) and control animals (**d-f**). Merged images correspond to Panels **c** and **f**, respectively. Scale bar, 100 μm. Panels **g**-**i** show MHCII^+^ cells residing in the meninges surrounding the motor cortex of a blast-exposed animal (positive control). Merged image is shown in Panel **i**. Scale bar, 20 μm

It is well known that brain injury triggers the proliferation and activation of quiescent ramified microglia that transform into proinflammatory brain macrophages (M1) devoid of branching processes and with upregulated expression of MHCII and other surface molecules such as CD86, and Fcγ receptors [7]. The negligible presence of MHCII^+^ Iba1^+^ cells in the hippocampus (<1% of total Iba1^+^ cells) of blast-exposed animals (similar to controls) further confirms the lack of neuroinflammation induced by the blast waves 6 weeks post-exposure (Fig. 3).

### Limited alterations in plasma or brain inflammasome after low-level blast exposure

**Fig. 4:**
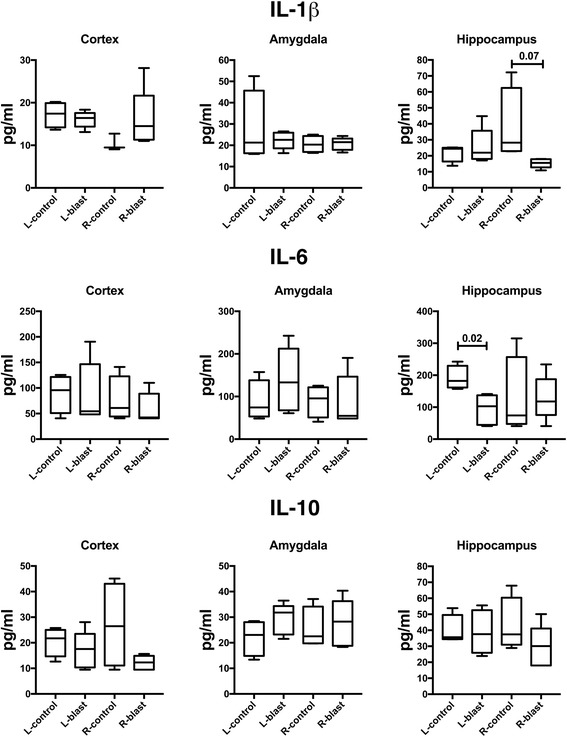
Selected cytokines in the brains of control and blast-exposed animals (6 weeks post-blast exposure). Shown are levels of IL-1β, IL-6 and IL-10 in the left (L) or right (R) hemispheres of the indicated brain regions. Error bars indicate the standard error of the mean (SEM). Statistical differences indicated represent unpaired *t*-tests

**Fig. 5:**
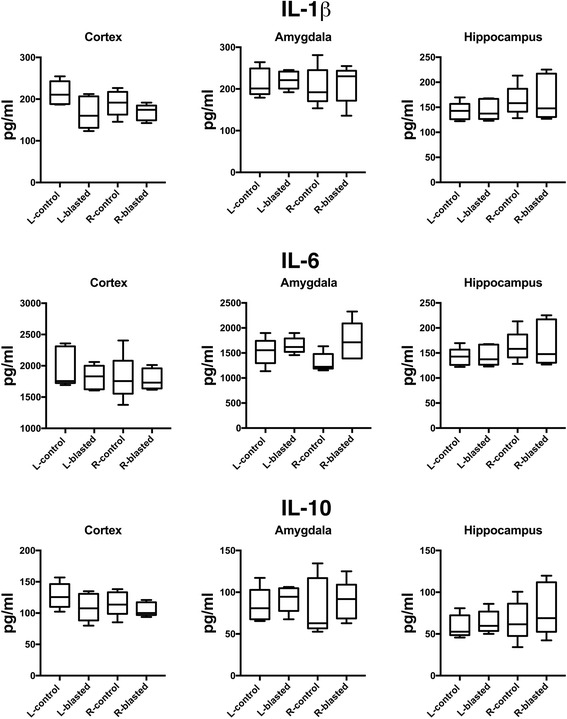
Selected cytokines in the brains of control and blast-exposed animals (40 weeks post-blast exposure). Shown are levels of IL-1β, IL-6 and IL-10 in the left (L) or right (R) hemispheres of the indicated brain regions. Error bars indicate the standard error of the mean (SEM). Statistical differences indicated represent unpaired *t*-tests

We also investigated the effects of low-level blast overpressures on the cerebral inflammasome in the amygdala, hippocampus, anterior cortex (prefrontal, motor, somatosensory, and insular cortices), and posterior cortex (association, auditory, visual, and entorhinal cortices) of both hemispheres and in plasma at 6 weeks and 40 weeks post-blast exposure (Tables 1 and 2, Figs. 4 and 5). As shown in Table 1, at 6 weeks post-blast exposure there were significant or near significant 1.4- to 1.8-fold decreases in the levels of fractalkine, IL-1β, leptin, LIX, MIP-1, and VEGFα in plasma. However, none of these changes was replicated in plasma at 40 weeks post-blast, where no cytokine differences between blast and control were detected (Fig. 5, Table 2). In brain, isolated changes were seen in selected cytokines at 6 weeks following blast exposure. However, none of these changes was replicated in both hemispheres (Table 1) or at 40 weeks post-blast exposure (Table 2). In addition, if a Bonferroni correction was applied for multiple comparisons (using *p* = 0.0002 for significance) none of the comparisons would reach statistical significance. Interestingly, as found in plasma in most cases where differences in brain were noted, the levels were decreased in blast-exposed animals. For example, as shown in Figs. 4 and 5, the proinflammatory IL-1β and IL-6 as well as the anti-inflammatory IL-10 were not significantly changed in the various brain areas except for a decrease of IL-6 in the right hippocampus. LIX was also decreased in the amygdala and right anterior cortex (Table 1). However, at 40 weeks there were no significant changes in the same cytokines in brain (Table 2). Collectively, these data provide little evidence for significant brain inflammation over a period of 6–40 weeks post-blast.

**Table 1:** Changes in cytokine/chemokine levels in plasma and in different brain regions as a consequence of blast exposure, measured at 6 weeks post-blast exposure

	L-Hipp	R-Hipp	L-Amy	R-Amy	L-AC	R-AC	L-PC	R-PC	Plasma
EGF	ND	ND	ND	ND	ND	ND	ND	ND	ND
Eotaxin	ND	ND	ND	ND	ND	ND	ND	ND	ND
Fractalkine	NC	NC	NC	NC	NC	1.4↓*p* = 0.08	1.5↓*p* = 0.01	NC	1.6↓*p* = 0.004
G-CSF	NC	NC	NC	NC	NC	NC	NC	NC	ND
GM-CSF	NC	NC	NC	NC	NC	NC	NC	NC	ND
GRO/KC	NC	NC	NC	NC	NC	NC	NC	1.4↑*p* = 0.008	ND
IFNγ	NC	NC	NC	NC	NC	NC	NC	NC	ND
IL-10	NC	NC	NC	NC	NC	NC	NC	NC	ND
IL-12p70	NC	NC	NC	NC	NC	NC	NC	NC	NC
IL-13	2.2↓*p* = 0.07	NC	NC	ND	NC	ND	ND	ND	NC
IL-18	1.9↓*p* = 0.03	NC	NC	NC	NC	1.4↓*p* = 0.09	1.7↓*p* = 0.02	NC	ND
IL-1α	1.5↓p = 0.02	NC	NC	NC	NC	2.5↓*p* = 0.09	NC	NC	NC
IL-1β	NC	2.5↓*p* = 0.07	NC	NC	NC	NC	NC	NC	1.8↓*p* = 0.048
IL-2	NC	NC	NC	NC	NC	NC	NC	1.7↑*p* = 0.02	NC
IL-4	NC	NC	NC	NC	NC	NC	NC	NC	NC
IL-5	1.5↓*p* = 0.02	NC	NC	NC	ND	ND	ND	ND	NC
IL-6	1.8↓*p* = 0.02	NC	NC	NC	NC	NC	ND	ND	NC
IL-17A	ND	ND	ND	ND	ND	ND	ND	ND	ND
IP-10	NC	NC	NC	NC	NC	NC	NC	NC	NC
Leptin	NC	1.8↓*p* = 0.01	ND	NC	NC	NC	NC	1.9↑*p* = 0.02	1.5↓*p* = 0.061
LIX	NC	NC	5↓*p* = 0.001	10↓*p* = 0.08	ND	11.2↓*p* = 0.02	ND	ND	1.8↓*p* = 0.006
MCP-1	1.2↓*p* = 0.09	NC	NC	NC	NC	ND	NC	NC	NC
MIP-1α	NC	NC	NC	ND	ND	ND	ND	ND	1.6↓*p* = 0.011
MIP-2	NC	NC	NC	NC	NC	NC	NC	NC	ND
RANTES	ND	ND	NC	ND	ND	NC	ND	ND	NC
TNFα	1.4↓*p* = 0.08	NC	NC	NC	NC	NC	NC	1.5↑*p* = 0.005	ND
VEGF	NC	NC	NC	NC	NC	ND	ND	ND	1.4↓*p* = 0.007

Up or down arrows indicate increased or decreased relative levels in blast-exposed versus control animals. The respective *p* value (unpaired *t*-tests) is also indicated. *NC*, no change, *ND* = not detected. *L* or *R* indicate left or right subregion, respectively. *Hipp* = Hippocampus, *Amy* = Amygdala, *AC* = Anterior cortex, *PC* = Posterior cortex

**Table 2:** Changes in cytokine/chemokine levels in plasma and in different brain regions as a consequence of blast exposure, measured at 40 weeks post-blast exposure

	L-Hipp	R-Hipp	L-Amy	R-Amy	L-AC	R-AC	L-PC	R-PC	Plasma
EGF	NC	NC	NC	NC	NC	NC	NC	NC	NC
Eotaxin	NC	NC	NC	NC	NC	NC	NC	NC	NC
Fractalkine	NC	NC	NC	NC	NC	NC	NC	NC	NC
G-CSF	NC	NC	NC	NC	NC	NC	NC	NC	NC
GM-CSF	NC	NC	NC	NC	NC	NC	NC	NC	NC
GRO/KC	NC	NC	NC	NC	NC	NC	NC	NC	NC
IFNγ	NC	NC	NC	NC	NC	NC	NC	NC	NC
IL-10	NC	NC	NC	NC	NC	NC	NC	NC	NC
IL-12p70	NC	NC	NC	NC	NC	NC	NC	NC	NC
IL-13	NC	NC	NC	NC	NC	NC	NC	NC	NC
IL-18	NC	NC	NC	NC	NC	NC	NC	NC	NC
IL-1α	NC	NC	NC	NC	1.3↓*p* = 0.03	NC	NC	NC	NC
IL-1β	NC	NC	NC	NC	NC	NC	NC	NC	NC
IL-2	NC	NC	NC	1.3↑*p* = 0.06	NC	NC	NC	NC	NC
IL-4	NC	1.3↑*p* = 0.06	NC	NC	NC	NC	NC	NC	NC
IL-5	NC	NC	NC	NC	1.3↓*p* = 0.04	NC	NC	NC	NC
IL-6	NC	NC	NC	1.3↑*p* = 0.06	NC	NC	NC	NC	NC
IL-17A	NC	NC	NC	NC	NC	NC	NC	NC	ND
IP-10	NC	NC	NC	NC	NC	NC	NC	NC	NC
Leptin	NC	NC	NC	NC	1.2↓*p* = 0.04	NC	1.2↓*p* = 0.04	NC	NC
LIX	NC	NC	NC	NC	NC	NC	NC	NC	NC
MCP-1	NC	NC	NC	NC	NC	NC	NC	NC	NC
MIP-1α	NC	NC	NC	NC	NC	NC	NC	NC	NC
MIP-2	NC	NC	NC	NC	NC	NC	NC	NC	NC
RANTES	NC	NC	NC	NC	NC	NC	NC	NC	NC
TNFα	NC	NC	NC	NC	NC	NC	NC	NC	NC
VEGF	NC	NC	NC	1.3↑p = 0.03	NC	NC	NC	NC	NC

Up or down arrows indicate increased or decreased levels in blast-exposed versus control animals.The respective p value is indicated. *NC*, no change; *L* or *R* indicate left or right subregion, respectively. *Hipp*, Hippocampus; Amy, Amygdala; *AC*, Anterior cortex; *PC*, Posterior cortex

### Focal hemorrhage triggers microglial activation in the blast-exposed brain

**Fig. 6:**
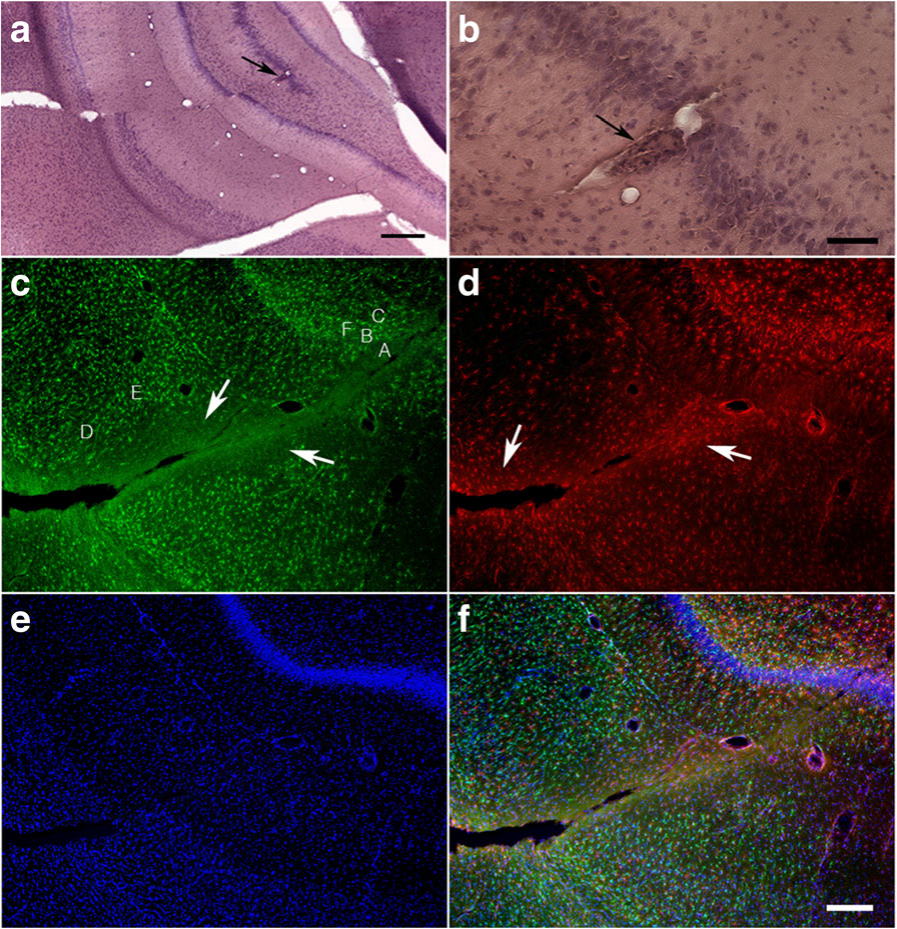
Focal tear and hemorrhage associated with microglia activation in the rat brain 16 weeks post-blast exposures. HE staining (**a**, **b**). Black arrows indicate location of a blood clot. Scale bars: (**a**), 500 μm; (**b**), 200 μm. Brain sections were stained with Iba1 (**c**) and GFAP (**d**) and counterstained with DAPI (**e**). Arrows in Panel (**c**) indicate the areas next to the focal tissue tear devoid of microglia. Letters inside Panel (**c**) indicate the relative location of areas illustrated in Fig. 7. Merged image (**f**). Scale bar, 200 μm

**Fig. 7:**
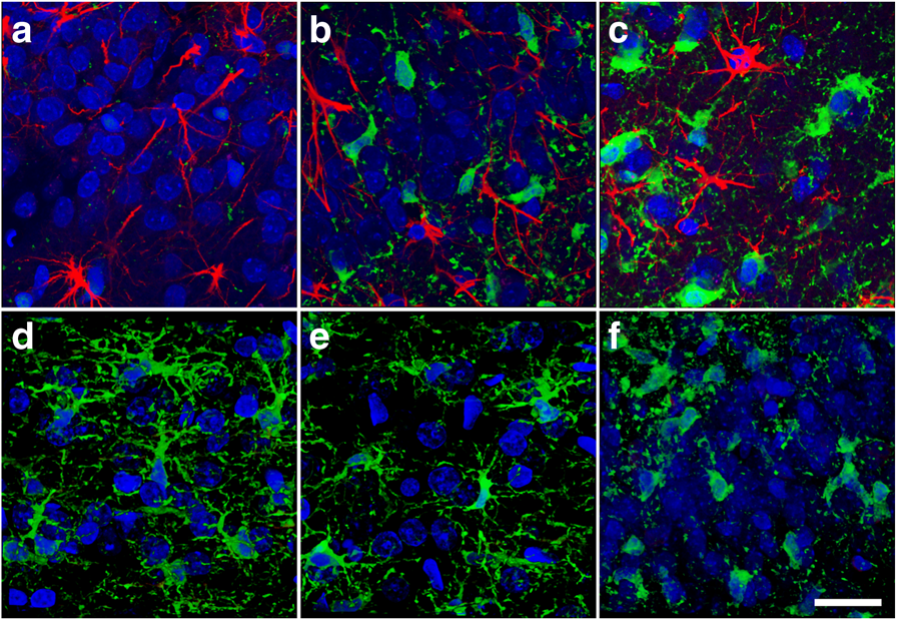
Distribution of microglia in the vicinity of a blast-induced hemorrhagic focal tear. The relative localization of the different panels in the brain of the blast-exposed animal is indicated in Fig. 6 (**e**). Panels (**a**-**c**), Iba 1 (green) and GFAP (red) immunostaining. Absence of microglia (green) in the molecular layer of the dentate gyrus immediately next to the blast-induced tear (**a**). Presence of reactive and ameboid microglia in the molecular layer of the dentate gyrus (**b**) and in the stratum lucidum (**c**) of the hippocampus, away from the tear. Microglial activation gradient, Iba 1 (green) immunostaining (**d**-**f**). Primed and reactive (types 2–3) microglia in the cortex (**d**, **e**). Ameboid (type 4) microglia in the region associated with the molecular layer of the dentate gyrus away from the tear (**f**). Scale bar, 50 μm

Interestingly, at 16 weeks post-blast exposure (3 × 74.5 kPa), the brain of a 6-month-old animal (part of a different cohort previously reported [49]) exhibited an amorphous cellular mass resembling an infiltrated clot within a scar tissue-tear associated with the perirhinal vein and presented with diffuse microgliosis within the injured hemisphere (Fig. 6). Microgliosis presented a gradient of reactivity including ameboid morphologies (types 3 and 4) that extended along the scarred lesion from the temporal association cortex through the CA1 stratum radiatum where the clot was found (Figs. 6 and 7). The scar of the focal tear was lined in its immediate vicinity by activated astrocytes and an area devoid of microglia (Figs. 6c-f, 7a), indicating that the local microglia are highly susceptible to the initial blast overpressure experienced in the region immediately adjacent to a penetrating vessel. This region was adjoined by an area of clear type 3 and 4 microgliosis. Type 4 ameboid microglia were predominantly found immediately next to the clot, whereas most cells in the cortical region exhibited the morphologies of types 2 and 3 activated cells (Figs. 6 and 7).

## Discussion

Acute inflammation is a recognized feature following TBI induced by mechanical trauma [47] with recent evidence that a chronic neuroinflammatory component may also develop and contribute to the late effects of TBI including the development of neurodegnerative diseases [14]. Inflammation is also a component of acute blast injury [12]. Activation of resident microglia, influx of peripheral leukocytes, and release of proinflammatory mediators are early events of this process. Microglia are activated by thrombin through MAPK signaling pathways via the proteinase-activated receptor-1 (PAR-1) [16, 39]. Microglia can also be activated through Toll-like receptors (TLRs) and the receptor for advanced glycosylation endproducts via danger-associated molecular signals (ATP, neurotransmitters, nucleic acids, heat shock proteins, and high-mobility group box 1 proteins) released by necrotic cells [10, 40, 52]. Microglial activation leads to increased production of TNF-α and IL-1β, which induces neuronal apoptosis [57]. Microglia are also involved in clearance of cell debris and phagocytosis of blood components, with a central role in hematoma resolution.

Explosive blasts rapidly generate very high levels of kinetic energy that dissipate as supersonic pressure waves to cause blast-induced TBI [13, 37, 42]. In the brain parenchyma, these high-energy, high-velocity blast waves can also cause substantial damage to blood vessels as well as to neuronal and glial cell bodies and their processes [4–6, 17, 19, 28, 49]. Prolonged but not short-duration high-energy blast waves (620–1570 kPa) result in the acute onset of neuroinflammation and of increased levels of pro-inflammatory cytokines in the brain [9]. Depending on the intensity of the blast, TBI may include an early-onset diffuse cerebral edema and delayed vasoconstriction [3, 34–36]. Injury secondary to blast-induced TBI involves vascular remodeling, neuroinflammation, and gliosis that are visible several months after the initial injury [6, 28, 37, 51].

In contrast to these findings after high-energy blast exposures, our experiments with lower level energy blast exposures (74.5 kPa) did not demonstrate the presence of chronic neuroinflammation 6 weeks post-blast exposure. Immunohistochemical analyses of brains from blast-exposed animals without any evidence of vascular leakage did not show obvious microgliosis, as shown by the relatively low abundance of Iba1^+^ reactive or amoeboid microglia (types 3 and 4) expressing MHCII, and did not present major alterations in the brain inflammasome even at 40 weeks post-blast exposure. Curiously, lack of inflammation after mild brain injury has also been reported in a mouse model of closed head injury using a standardized weight-drop technique [45]. The lack of inflammation observed in our animals indicates that low-energy blast exposures (74.5 kPa) are not always sufficient to sustain chronic neuroinflammation. In a murine model system, microglial activation associated with microdomains of vascular disruption (tight junction injury) has been observed 45 min post 105.5-kPa blast exposure [22]. However, by 14 days post-blast, elevated levels of TNF-α were only sustained in animals exposed to three repetitive blasts, suggesting that even at higher blast energy, repetitive exposures are required to promote more persistent neuroinflammatory changes in the CNS [22].

In blast-induced TBI, vascular blood leakage may be a requirement for the progression and persistence of neuroinflammation. This is also supported by an observation in a blast-exposed animal that exhibited an infiltrated clot within scarred tissue 16 weeks post-blast exposure that was associated with overwhelming microglial activation (Figs. 6 and 7). As the clot was found 16 weeks post-blast exposure, it could be presumed that the vascular leakage occurred at a much later time after the last blast exposure, most likely as a result of a progressive blast-induced vascular degenerative processes, leading to induced vascular fragility, subsequent rupture, and blood leakage [17]. We have previously reported the selective vulnerability of penetrating vessels that follow the patterns of blast-induced focal tears and of the associated microvasculature to 74.5-kPa blast exposures. Vascular injuries are present acutely at 24 h after blast exposure and lead to a chronic vascular degenerative processes that can lead to vascular rupture later in life [17].

Comparison of high- and lower-energy blast-induced TBI clearly indicates that BBB leakage, microglial activation, and increased production of pro-inflammatory cytokines depend on the blast overpressure energy and the number of exposures. Acutely, after 72 h, animals exposed to 74.5-kPa blasts present pathological alterations that mainly included blood leakage from the choroid plexus into the lateral ventricles, focal non-hemorrhagic tissue tears, and vascular alterations [17, 49]. In other rat models, blast-induced BBB leakage appeared preferentially at higher blast pressures (>110-kPa), as it was shown that extensive leakage occurred in all brain regions but preferentially in the thalamus, striatum, hippocampus, and occipital cortex [25, 29]. However, limited leakage, mainly through the chorionic plexus, was observed after exposure to 72-kPa blasts [25, 29, 49]. Kawoos et al. [29] also showed that there is a qualitative relationship between BBB leakage and increased intracerebral pressure (ICP), through which the ICP levels and sustainability depend also on blast intensity and the number of blast exposures. As blast induces an early vasodilation (as evidenced by enlarged blood vessel diameters [25]), the intravascular forces exercised by the pressurized circulating blood could damage the vascular smooth muscle and endothelial layers [2, 17, 19, 20, 25, 29, 49, 56]. Through buckling, it could induce vascular tortuosity [21] and through subsequent axial stretch [15], generate vascular strictures [17, 49]. In small vessels, endothelial damage can also lead to a breakdown of the BBB and blood leakage [25, 29]. In large vessels, endothelial damage could trigger chronic vascular disease through vascular remodeling by induction of neointima formation, neointima thickening, restenosis, aneurysm formation, plaque deposition, vascular occlusion, thromboembolism, and vascular rupture. Therapeutic approaches aimed at preventing or reversing vascular damage may improve the chronic neuropsychiatric symptoms associated with blast-induced TBI.

## Conclusions

Recently much interest has been generated in the role of ongoing neuroinflammation in the chronic effects of TBI. We investigated whether chronic neuroinflammation was present in a rat model of repetitive low-energy blast exposure. No significant alterations in the levels of neuroinflammatory indicators (microglia proliferation and activation as well as pro-inflammatory cytokine/chemokine concentrations) were observed in rats 6-40 weeks after three 74.5-kPa blast exposures. Microgliosis and microglial activation were observed in one animal associated with vascular blood leakage 16 weeks post-blast, most likely due to chronic vascular degeneration. Thus, extravasation of blood elements may be a trigger for neuroinflammation in blast-induced TBI. However in the absence of hemorrhage, chronic neuroinflammation does not appear to be a long-term consequence of low-level blast injury.
